# Decreased Basal Ganglia Activation in Subjects with Chronic Fatigue Syndrome: Association with Symptoms of Fatigue

**DOI:** 10.1371/journal.pone.0098156

**Published:** 2014-05-23

**Authors:** Andrew H. Miller, James F. Jones, Daniel F. Drake, Hao Tian, Elizabeth R. Unger, Giuseppe Pagnoni

**Affiliations:** 1 Department of Psychiatry and Behavioral Sciences, Emory University School of Medicine, Atlanta, Georgia, United States of America; 2 Chronic Viral Diseases Branch, Centers for Disease Control and Prevention, Atlanta, Georgia, United States of America; 3 Department of Neuroscience, Biomedical, Metabolic Sciences, Università Degli Studi Di Modena E Reggio Emilia, Modena, Italy; Centre national de la recherche scientifique, France

## Abstract

Reduced basal ganglia function has been associated with fatigue in neurologic disorders, as well as in patients exposed to chronic immune stimulation. Patients with chronic fatigue syndrome (CFS) have been shown to exhibit symptoms suggestive of decreased basal ganglia function including psychomotor slowing, which in turn was correlated with fatigue. In addition, CFS patients have been found to exhibit increased markers of immune activation. In order to directly test the hypothesis of decreased basal ganglia function in CFS, we used functional magnetic resonance imaging to examine neural activation in the basal ganglia to a reward-processing (monetary gambling) task in a community sample of 59 male and female subjects, including 18 patients diagnosed with CFS according to 1994 CDC criteria and 41 non-fatigued healthy controls. For each subject, the average effect of winning vs. losing during the gambling task in regions of interest (ROI) corresponding to the caudate nucleus, putamen, and globus pallidus was extracted for group comparisons and correlational analyses. Compared to non-fatigued controls, patients with CFS exhibited significantly decreased activation in the right caudate (p = 0.01) and right globus pallidus (p = 0.02). Decreased activation in the right globus pallidus was significantly correlated with increased mental fatigue (r^2^ = 0.49, p = 0.001), general fatigue (r^2^ = 0.34, p = 0.01) and reduced activity (r^2^ = 0.29, p = 0.02) as measured by the Multidimensional Fatigue Inventory. No such relationships were found in control subjects. These data suggest that symptoms of fatigue in CFS subjects were associated with reduced responsivity of the basal ganglia, possibly involving the disruption of projections from the globus pallidus to thalamic and cortical networks.

## Introduction

There has been increasing interest in the neural correlates of fatigue and other symptoms that afflict individuals with chronic fatigue syndrome (CFS). CFS has devastating effects on social and occupational function, leading to significant personal and public health costs [1]–[4]. One potential focus for the neurocircuitry of fatigue in CFS is the basal ganglia. The basal ganglia play a fundamental role in the regulation of motor activity and motivation and have been implicated as a major brain system associated with fatigue [5]. For example, fatigue is common in patients with neurologic disorders that involve the basal ganglia including Parkinson's disease and multiple sclerosis as well as in human immunodeficiency virus (HIV), where infected patients exhibit early cognitive/motor disturbances [5]–[9]. In addition, lesions of the basal ganglia have been associated with marked fatigue as well as psychomotor slowing [5]–[7]. In CFS patients, neurocognitive testing has also revealed specific impairments compatible with basal ganglia dysfunction, including motor slowing that correlated with severity of fatigue [10]. In addition, using magnetic resonance spectroscopy, CFS patients have shown increased basal ganglia choline concentrations, possible evidence of increased inflammation (glial activation) and/or ischemia [11]. Of note, increased peripheral blood markers of inflammation have been reported in CFS subjects [12]–[15], and chronic fatigue is often the outcome of a number of viral infections [16]–[18]. Interestingly, administration of inflammatory stimuli has been shown to alter basal ganglia function in association with fatigue [19]. Indeed, using functional magnetic resonance imaging (fMRI), chronic administration of the anti-viral and inflammatory cytokine interferon alpha was shown to reduce the neural response of the basal ganglia (ventral striatum) to a hedonic reward task [19], and changes in neural activity were in turn highly correlated with symptoms of fatigue [19]. Similar results have been found after acute administration of endotoxin to healthy volunteers where the basal ganglia response to hedonic reward was found to be reduced [20]. Taken together, these data suggest that inflammatory stimuli including viruses, cytokines and cytokine inducers can cause fatigue through alterations in basal ganglia function.

In order to further explore basal ganglia function in patients with CFS and its relationship with CFS-related symptoms, we conducted a fMRI study in CFS patients to examine the basal ganglia response to hedonic reward during a gambling task previously shown to activate relevant basal ganglia nuclei [21]. The correlation of the observed degree of neural activation in relevant basal ganglia nuclei with symptoms of fatigue was also assessed.

## Materials and Methods

### Ethics Statement

The study was approved *a priori* by the Emory and Centers for Disease Control and Prevention Institutional Review Boards. All subjects provided written informed consent prior to study participation.

### Subjects

Participants in this study represent a subgroup of persons enrolled in the baseline and follow-up survey of CFS in Georgia [1]. In brief, the baseline survey took place in 2004–2005 and used random-digit-dialing screening interview followed by detailed phone interview to identify participants who met interview criteria for CFS (CFS-like), those who were well (control), or those with some CFS case-defining symptoms (intermediate group). All CFS-like participants, controls (matched to CFS-like on age, sex, race and geographic strata), and a similar number of randomly selected participants from the intermediate groups were invited for clinical evaluation. The clinical visit included detailed medical history, physical examination, laboratory tests, psychiatric screen, and questionnaires to measure functional status, impairment and symptoms, and was completed by 783 persons. Participants were classified as: i) CFS cases, according to the 1994 case definition [22], [23]; ii) non-fatigued controls; iii) insufficient symptoms or fatigue for CFS; or iv) exclusionary medical or psychiatric condition. Demographic information, including education, was collected during the telephone interview and verified in the clinic. Follow-up occurred during 2007–2009 and included all baseline participants without permanent exclusionary conditions seen in clinic (n = 681) as well as those completing detailed telephone interview (n = 3049). Participants were re-interviewed by phone and invited for follow-up clinical examination. Participants in the baseline detailed telephone interview who had no exclusions were also contacted again and re-interviewed as part of the follow-up. The follow-up clinic evaluated 751 participants and classified them in the manner described above. Following clinical and laboratory assessments, 71 persons met criteria for CFS, and 212 were determined to be controls. Medical and psychological exclusions for CFS include history of head trauma or seizures; unstable cardiovascular, endocrine, hematologic, renal or neurologic disease (determined by physical examination and laboratory testing); hepatitis B or C or human immunodeficiency virus infection (by medical history); current or past history of schizophrenia or bipolar disorder or a current diagnosis of major depression [as determined by Structured Clinical Interview for Diagnostic and Statistical Manual of Mental Disorders–Fourth Edition (SCID)][24]. Subjects with anxiety disorders were not excluded. Subjects between the ages of 18 and 60 years of age who met criteria for the fMRI study were then invited to the Emory General Clinical Research Center for a fMRI scanning session as described below. The sample was recruited on a 1∶2 ratio basis (CFS:control).

### Exclusion Criteria for fMRI

Exclusion criteria for all subjects included: left-handedness (to minimize the variance associated with the effect of handedness on brain structure and function); presence of metal in the body; claustrophobia; and a reading level below grade 7 (as determined by the WRAT 3, to assure comprehension of written instructions during the gambling task). Subjects with a score >60 on the 20-item, self report Zung Self Rating Depression Scale (Zung SDS)(indicating more than mild depressive symptoms) were also excluded [25]. All subjects were required to be free of psychotropic medications including antidepressant, antipsychotic, mood stabilizer or anti-anxiety medications for at least 4 weeks prior to brain imaging procedures. No subjects were taken off psychotropic medications for the purposes of the study. Other medications (see Table 1) were not exclusionary. Subjects with a history of alcohol/psychoactive substance abuse or dependence within the past year, as determined by SCID, were excluded. Urine drug screens were conducted prior to all brain scans to rule out active substance abuse.

**Table 1:** Sociodemographic and Clinical Characteristics of the Study Sample.

Characteristic	Control (n = 41)	CFS (n = 18)	p value
Age (yrs.) – mean (SD)	47.2 (9.2)	44.2 (11.1)	0.28
Sex (female) – no. (%)	33 (80.0%)	16 (88.9%)	0.71
Ethnic Origin - no. (%)			0.48
Caucasian	35 (85.4%)	14 (77.8%)	
Black	6 (14.6%)	4 (22.2%)	
Education (Highest Degree) – no. (%)			0.66
Graduate Degree	11 (26.8%)	7 (38.9%)	
College Graduate	9 (22.0%)	5 (27.8%)	
Partial College	16 (39.0%)	4 (22.2%)	
High School Graduate	4 (9.8%)	1 (5.6%)	
Junior High School	1 (2.4%)	1 (5.6%)	
BMI (kg/m^2^) – mean (SD)	26.7 (5.1)	28.1 (4.6)	0.35
Zung SDS – mean (SD)	32.5 (4.5)	51.7 (9.4)	<0.0001*
SF-36 – mean (SD)			
Bodily Pain	89.0 (11.3)	49.5 (17.7)	<0.0001*
General Health	91.1 (7.6)	52.6 (21.5)	<0.0001*
Mental Health	92.6 (5.2)	68.2 (18.1)	<0.0001*
Physical Functioning	96.5 (5.4)	64.4 (25.9)	<0.0001*
Role-Emotional	99.2 (5.2)	64.8 (45.0)	<0.0005*
Role-Physical	99.4 (3.9)	51.4 (45.8)	<0.005*
Social Functioning	98.8 (6.1)	67.4 (22.7)	<0.0001*
Vitality	86.3 (9.6)	40.3 (20.3)	<0.0001*
CDC Symptom Inventory – mean (SD)			
CFS Symptoms	0.4 (0.7)	4.6 (1.8)	<0.0001*
Total Symptoms (CFS + non-CFS)	1.1 (1.4)	8.1 (3.9)	<0.0001*
CFS Case Definition Subscale	1.1 (2.3)	37.4 (23.0)	<0.0001*
Medication Usage – no (%)			
Anti-inflammatory	11 (27%)	6 (33%)	0.76
Hormonal	15 (37%)	6 (33%)	1.00
Analgesic^a^	3 (7%)	3 (17%)	0.36
Gastrointestinal	9 (22%)	6 (33%)	0.52
Anti-Allergy	12 (29%)	6 (33%)	0.77
Cardiovascular^b^	14 (34%)	6 (33%)	1.00
Supplements^c^	23 (56%)	7 (39%)	0.27
Sleep Aids	1 (2%)	1 (6%)	0.52

yrs-years; SD-standard deviation; no.-number; BMI-body mass index; Zung SDS-Zung Self Rating Depression Scale; SF-36: Short-Form (36) Health Survey; CFS-Chronic Fatigue Syndrome;

*- Welch Test;

a - one subject in each group used narcotic analgesics;

b - anti-hypertensive medications and cholesterol lowering medications;

c - vitamins and herbal preparations.

### fMRI Imaging Task

A previously published gambling task, proven effective in eliciting specific activation of basal ganglia structures, was adapted for the study [19], [21]. In the task, participants had to guess which of two cards presented face-down on a screen was “red” (hearts or diamonds) by pressing one of two buttons on a MRI response box held in their right hand. Two seconds into the trial, the selected card was turned over, and, depending on its color, the participant either won (red card) or lost (black card) one dollar. Unbeknownst to the subject, the sequence of wins and losses was temporally arranged as a noisy sinusoid with a slow linear trend that favored wins over time. This procedure allowed for experimental control of the task while masking the deterministic nature of the game from the participant, thereby eliciting a realistic feeling of gambling while playing. At the beginning of the game, each participant started with a credit of $16 and ended with a total win sum of $23, which the volunteer believed was contingent on his/her specific gambling choice but was in fact fixed for all subjects.

### fMRI Processing and Analyses

Three T2*-weighted functional runs [gradient echo-planar imaging (EPI), TR: 2.35 s, TE: 28 ms, 135 volumes/run, voxel size: 3 mm isotropic, 64×64 voxel matrix, 35 slices] and a high-resolution T1-weighted anatomical scan (MPRAGE, TR: 2.3 s, TE: 3.02 ms, 1 mm isotropic voxels, whole-brain coverage) were collected on a Siemens Trio 3T MRI scanner. All fMRI processing was performed using the Analysis of Functional NeuroImage (AFNI) software package [26]. For each subject, the three EPI runs were corrected for slice-acquisition timing and aligned via a rigid-body transformation to a common reference EPI volume to minimize artifacts from head motion. The anatomical volume was co-registered to the EPI reference volume and then spatially normalized to the Talairach-Tournoux standard stereotaxic brain space. The same transformation was used to warp all functional volumes to standard space. Each volume was smoothed with a 4 mm-FWHM Gaussian kernel and intensity-normalized to a percent change relative to the run's temporal mean on a voxel-wise basis. Task-related activation maps were computed for each subject using AFNI's Restricted Maximum Likelihood procedure that minimizes the temporal auto-correlation of the scans prior to ordinary least squares model fit. The model matrix was block-diagonal, one block per run. Each block contained (a) a baseline model of four Legendre polynomials (degree zero to three) plus six motion parameters, to account for signal drifts and head motion residual confounds, and (b) two task regressors modeling the trials when a red card (Win) and a black card (Lose) were selected respectively, obtained by convolving the corresponding stimulus timing vectors with a standard hemodynamic gamma function. General linear tests were used to compute the Win-Lose contrast combined over all three runs.

To focus the analysis on the specific basal ganglia regions activated by the task, the following procedure was employed: (1) a whole-brain group analysis revealing the general activation pattern for the Win-Lose contrast across all subjects was performed; (2) the resultant statistical parametric brain map thresholded at p<0.05, corrected for multiple comparisons, was intersected with basal ganglia regions of interest (ROIs: caudate nucleus, putamen, and globus pallidus), obtained from a probabilistic cytoarchitectonic brain atlas included in the AFNI distribution; (3) for each subject, the average value of Win-Lose activation contrast in each ROI was extracted for group comparisons and correlational analyses.

### Illness Assessment

To determine the status of participant's illness at the time of the scan, questionnaires were administered the evening before the scan. Fatigue was assessed using the 20-item Multidimensional Fatigue Inventory (MFI) 20, a self-rating scale yielding specific subscales for general fatigue, physical fatigue, reduced activity, reduced motivation and mental fatigue, with the summed score for each subscale ranging from 4 (best) to 20 (worst)[27]. Perceived health and function was measured by the Short Form (36) Health Survey (SF-36), a self-rating tool that provides eight subscale scores including bodily pain, general health perceptions, mental health, physical functioning, emotional role functioning, physical role functioning, social role functioning and vitality. Higher scores indicate better perceived health and function. Symptoms of CFS were assessed by the CFS Symptom Inventory, which is a self-report instrument that collects information about the presence, frequency and intensity of fatigue and 18 illness-related symptoms over the past month period [28], [29]. Finally, symptoms of depression were measured by the Zung Self-rating Depression Scale (SDS), a 20-item self-report scale which measures the presence of a variety of depressive symptoms including depressed mood, anhedonia, concentration problems, sleep difficulties, fatigue, self-esteem, bodily complaints and suicidal ideation over the past several days. Higher scores indicate greater severity of depressive symptoms [25].

### Statistical Analysis

Differences between groups in sociodemographic and clinical variables were assessed using t-tests for continuous measures and chi-square tests (or Fisher's Exact Test where appropriate) for categorical variables. In cases of unequal variances, Welch's t-test was employed. Evoked activity in right, left and bilateral caudate, putamen and globus pallidus was determined by averaging the estimated percent blood oxygen level-dependent (BOLD) signal change corresponding to the Win-Lose contrast across all ROI voxels as described above. Differences in overall neural activity between groups across ROIs in the three right and left basal ganglia nuclei (caudate, putamen and globus pallidus) were analyzed using a multivariate analysis of covariance (MANCOVA) controlling for age sex, race and body mass index (BMI) as well as the presence or absence of an anxiety disorder or the use of medications categorized by class (see Table 1). The impact of these covariates on neural activity in these basal ganglia nuclei of CFS subjects only was also assessed using multivariate regression analyses. Given that the omnibus F-test was significant, univariate analyses (t-tests) were then performed to investigate group differences in specific ROIs. Relationships between dimensions of fatigue as measured by the MFI and evoked activity in those ROIs that significantly differed between groups were assessed in each group separately using Bravais-Pearson correlations.

## Results

### Sociodemographic characteristics and clinical variables

Eighteen CFS patients and 41 healthy controls qualified for the study and completed the fMRI procedure. Subjects were well-matched on age, sex, race, education, BMI and medication status (Table 1). However, as expected, CFS patients exhibited significantly higher levels of depressive symptoms, lower perceived health and function and a greater number of CFS- and non-CFS defining symptoms (Table 1). Significantly greater scores for all dimensions of fatigue were also observed in the CFS group compared to controls (Table 2). Six CFS patients had a current diagnosis of one or more anxiety disorders (1 with obsessive compulsive disorder, 1 with post traumatic stress disorder, 2 with generalized anxiety disorder, and 4 with a phobic disorder. No control subject had a diagnosis of an anxiety disorder.

**Table 2:** Symptoms of Fatigue in CFS and Control Subjects.

MFI Subscale	Control (n = 41)	CFS (n = 18)	t statistic	p value*
	Mean (SD)	Mean (SD)		
General Fatigue	6.0 (1.6)	14.7 (3.2)	10.9	<0.0001
Physical Fatigue	5.6 (2.3)	12.2 (4.5)	5.8	<0.0001
Reduced Activity	5.0 (1.3)	10.4 (5.1)	4.4	<0.001
Reduced Motivation	5.3 (1.5)	10.2 (2.7)	7.2	<0.0001
Mental Fatigue	5.8 (1.8)	12.7 (3.8)	7.3	<0.0001

CFS-Chronic Fatigue Syndrome; MFI-Multidimensional Fatigue Inventory; SD-Standard deviation;

*-Welch test.

### Neural Activity in Response to the Gambling Task

**Figure 1:**
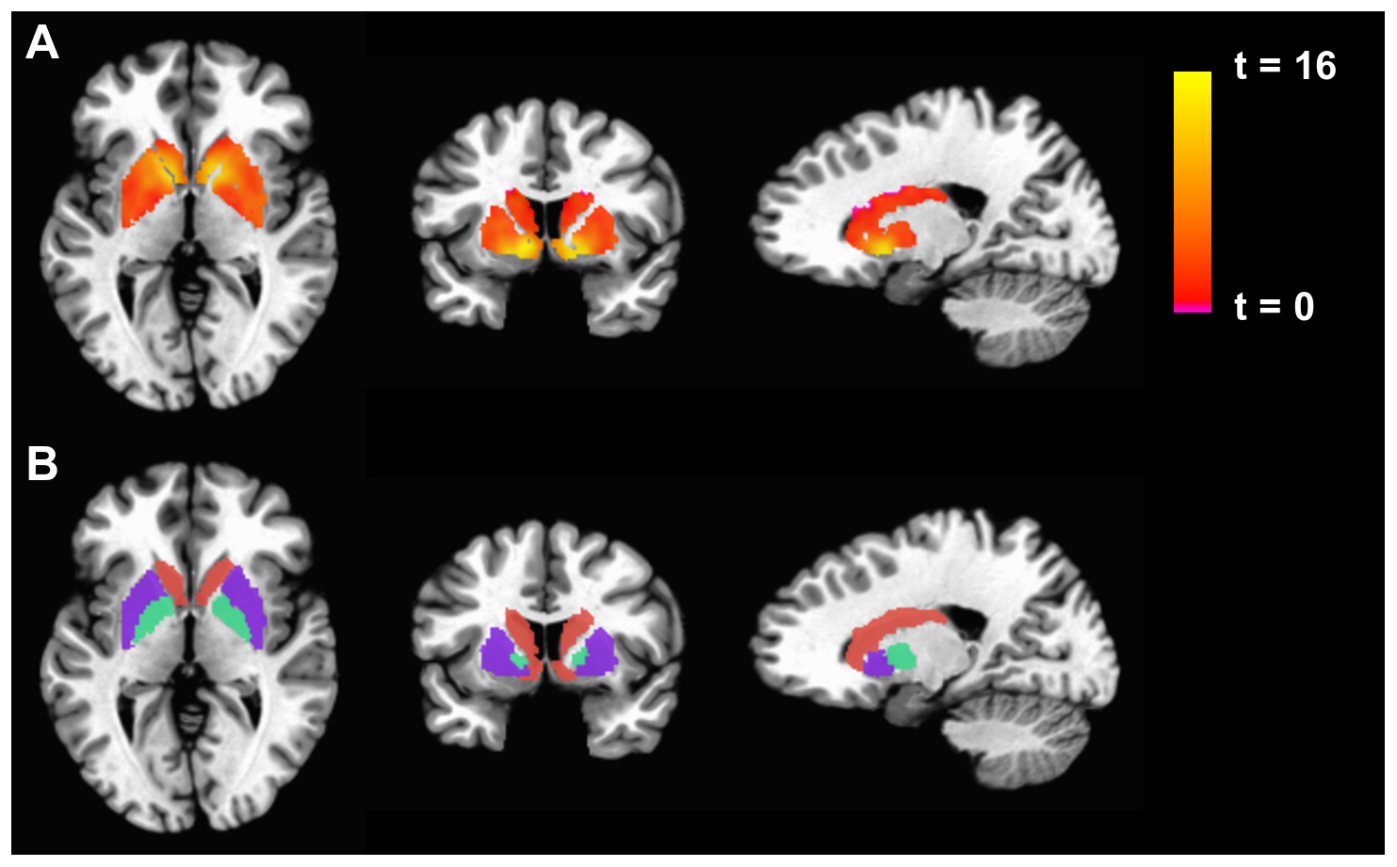
Basal Ganglia Activation in the Gambling Task. Left to right - Axial, coronal and transverse sections of the brain. The top row displays the activation for the Win-Lose contrast, for the pooled sample of chronic fatigue syndrome (CFS)+Control subjects, as a statistical parametric map thresholded at a p<0.05 corrected threshold and masked with the atlas-based anatomical regions of interest portrayed in the bottom row (putamen: purple; caudate: orange; globus pallidus: turquoise).

**Figure 2:**
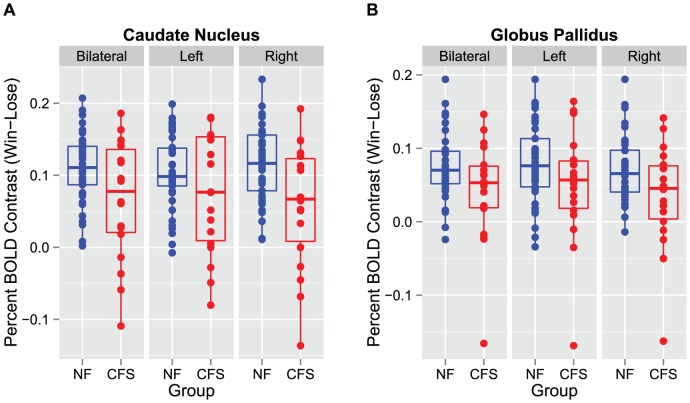
Reduced Activation in Basal Ganglia Structures in CFS compared to Controls for the Win-Lose Contrast. Box plot of activation [percent blood oxygen level-dependent (BOLD) signal change corresponding to the Win-Lose contrast averaged across all region of interest (ROI) voxels] in basal ganglia structures: blue  =  nonfatigued (NF) controls, red  =  chronic fatigue syndrome (CFS) subjects. Univariate analyses of group differences in specific ROIs revealed that CFS subjects exhibited significantly reduced neural activity compared to NF controls in the right caudate and right globus pallidus and each of these structures bilaterally (all p<0.05).

As shown in Figure 1, in the sample as a whole, there was a marked increase in neural activity in the Win-Lose condition as indicated by an increased BOLD signal throughout basal ganglia structures including the caudate, putamen and globus pallidus. Comparison of the neural activity between CFS and controls across the basal ganglia nuclei revealed a significant overall effect of group controlling for age, sex, race and BMI (F[1,53] = 2.9, p = 0.02). The overall group effect was also observed when controlling for the presence or absence of an anxiety disorder or when controlling for each category of medication individually (along with age, race, sex, and BMI) (all p<0.05). In addition, neural activity in the various basal ganglia nuclei of CFS subjects only was not significantly related to the presence or absence of an anxiety disorder (p = 0.48) or whether or not subjects were taking a medication from each of the individual categories indicated in Table 1 analyzed in conjunction with age, race, sex and BMI (all p>0.05). Univariate analyses revealed that CFS subjects exhibited significantly reduced activation for winning versus losing trials compared to controls in the right caudate (CFS: 0.057 ± SD 0.086 versus control: 0.116 ± SD 0.051, p = 0.014) and right globus pallidus (CFS: 0.035 ± SD 0.071 versus control: 0.079 ± SD 0.053, p = 0.019) (Figure 2). Significant decreases were also found in CFS subjects versus controls in the bilateral caudate nucleus (CFS: 0.066 ± SD 0.083 versus control: 0.110 SD ± 0.047, p = 0.047) and bilateral globus pallidus (CFS: 0.043 ± SD 0.071 versus control: 0.075 ± SD 0.045, p = 0.036), but these differences did not reach significance when the left side was considered alone (data not shown).

### Correlational Analyses between Symptoms and Basal Ganglia Activation

**Figure 3:**
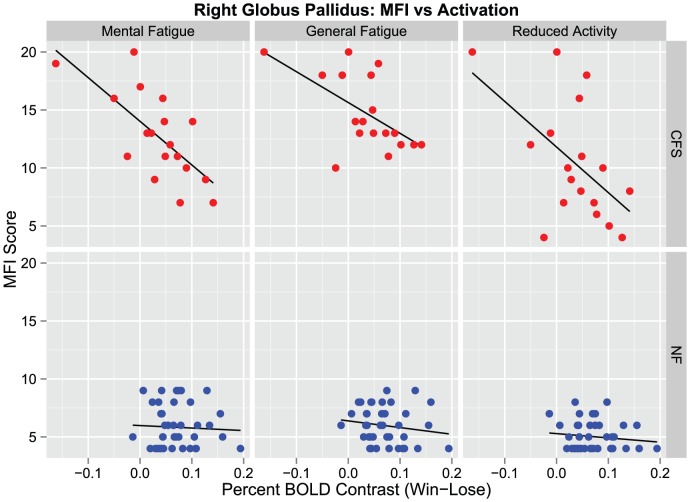
Correlation between Fatigue and Globus Pallidus Activation in CFS and Controls. Scores on the mental fatigue, general fatigue and reduced activity subscales of the Multidimensional Fatigue Inventory (MFI) are plotted against the percent BOLD signal change corresponding to the Win-Lose contrast averaged across all region of interest (ROI) voxels in the right globus pallidus: blue  =  nonfatigued (NF) controls, red  =  chronic fatigue syndrome (CFS) subjects. Relationships were assessed in each group separately using Bravais-Pearson correlations. Significant correlations were found between neural activity in the right globus pallidus and mental fatigue, general fatigue and reduced activity in CFS subjects (all p<0.05) but not in controls.

In CFS subjects, strong correlations were found between reduced evoked activity in the right globus pallidus and symptoms of fatigue, with the highest correlations occurring in association with mental fatigue (r^2^ = 0.491, p = 0.001), general fatigue (r^2^ = 0.338, p = 0.011) and reduced activity (r^2^ = 0.293, p = 0.02) subscales of the MFI (Figure 3). Similar significant relationships were observed for the physical fatigue subscale (r^2^ = 0.24, p = 0.04) but not for the reduced motivation subscale of the MFI (r^2^ = 0.03, p = 0.47). No significant correlations were found between symptoms of fatigue and the right caudate nucleus in CFS subjects. In addition, there were no significant correlations in control subjects between basal ganglia activation in any of the identified ROIs and fatigue symptoms (Table 2). The measured correlation coefficients between neural activity and fatigue in CFS subjects in the right globus pallidus were not significantly changed after controlling for age, sex, race and BMI (data not shown).

## Discussion

CFS subjects exhibited reduced neural activation to a reward task in caudate and globus pallidus, which in turn was correlated with symptoms of fatigue. These data suggest that basal ganglia circuits, especially those involving the globus pallidus, are associated with the expression of fatigue in CFS subjects. In addition, the data suggest that the neurocircuitry of fatigue in CFS patients may share a similar basis in the basal ganglia as is observed in other neurologic disorders and cases of basal ganglia lesions, as well as in the context of immune activation.

The observed reduction in the response of the globus pallidus to the gambling task which correlated with symptoms of fatigue in CFS patients is consistent with previous studies on this basal ganglia region. The pars interna of the globus pallidus (GPi), along with the substantia nigra pars reticulata, are main basal ganglia outputs to the thalamus and receive significant dopaminergic projections from the substantia nigra as well as inhibitory GABA input from the striatum [30], [31]. A loss of the dopaminergic input to the striatum, which characterizes Parkinson's disease, leads to the hyper-activity of the GPi and the subthalamic nucleus (STN)[32], [33], an observation that has led both nuclei to become targets for deep brain stimulation in the treatment of Parkinson's disease [34], [35]. DBS in Parkinson's disease primarily works by decreasing the abnormally-high neural transmission in the GPi or STN [36]. However, a potential side effect of DBS in Parkinson's disease is the onset of apathy which is believed to be secondary to decreased pallidal responsivity, a finding in agreement with our observation of increased symptoms of fatigue in the context of reduced pallidal responses to reward stimuli. Similarly, a consistent body of clinical data on cerebrovascular insults has associated apathy with a disruption of the normal activity of the GPi and its related thalamo-frontal and prefrontal circuits [37], [38], characterized by the inability to initiate thoughts or behaviors [39]. Finally, studies from the animal literature strongly implicate the globus pallidus in reward, motivation, and effort-related choice behavior [40]–[42], all processes that have been found to be critically affected in CFS patients. Thus, alterations in globus pallidus activation, possibly secondary to disruptions in neurotransmitter inputs involving GABA and dopamine, may contribute to symptoms of fatigue in patients with CFS. Of note, we observed a significant decrease of task-related activation in CFS patients compared to control subjects in the right caudate nucleus and in the right globus pallidus, where the amplitude of the effect in the latter structure was correlated with symptoms of fatigue. The potential cause for the right-hemisphere predominance of the findings is a matter of speculation. Small hemispheric asymmetries in morphometric measures of the basal ganglia have been previously reported in healthy subjects [43], as well as in patients with attention deficit disorder (ADHD), where a dysfunction of a right-sided striatal-prefrontal circuit has been proposed to underlie the attentional deficits [44]. Perhaps more relevant to our current findings, a similarly right-hemisphere biased effect as the one reported herein was observed in a PET study using a radioisotope of the dopamine precursor L-DOPA in patients with Parkinson's disease, with the degree of impaired dopamine uptake in the right caudate nucleus correlating significantly with the level of cognitive impairment [45]. Finally, an earlier study of neurotransmitters distributions in post-mortem human brains found that individuals with lower amounts of total dopamine tended to concentrate dopamine in the left putamen and globus pallidus, compared to their right-sided counterparts [46]. Although the precise role and meaning of hemispheric differences in basal ganglia function will need to be clarified by future larger and more focused studies, the latter finding supports the hypothesis of a general hypofunctional dopaminergic system in CFS that exposes the right basal ganglia structures to a greater vulnerability.

One mechanism that may contribute to alterations in function and neurotransmission in the basal ganglia in CFS is inflammation [47]. CFS patients have been shown to exhibit a number of immune alterations including the presence of increased inflammatory markers in the peripheral blood and increased production of inflammatory cytokines in ex vivo preparations of peripheral blood mononuclear cells [12]–[15]. As noted above, a number of inflammatory stimuli including the inflammatory cytokine, interferon alpha and cytokine inducers such as endotoxin and typhoid vaccination have been shown to alter basal ganglia function while also leading to symptoms of fatigue including psychomotor slowing and reduced motivation, both fundamental behavioral processes regulated by basal ganglia structures [19], [20], [47], [48]. Recent work in humans and non-human primates suggests that some of the effects of inflammatory stimuli, in particular interferon alpha, are mediated by effects on basal ganglia dopamine [19], [49]. Indeed, studies in humans using positron emission tomography have shown increased uptake and decreased turnover/release of the dopamine precursor, L-DOPA [19]. Similar results have been found in rhesus monkeys administered interferon alpha [49]. Using in vivo microdialysis from probes implanted in the caudate, dopamine release was found to be decreased after 4 weeks of interferon alpha, consistent with the reduced neural activation in basal ganglia nuclei seen in humans using fMRI in both interferon alpha-treated subjects as well as patients with CFS [19], [49]. Of note, interferon alpha is a cytokine well known to be released during viral infections, and increased levels of central nervous system interferon alpha have been associated with behavioral deficits in animal models of human immunodeficiency virus (HIV) and HIV patients [50], [51]. Thus, the activation of inflammatory pathways by viruses or other pathogens – as well as in a variety of conditions known to be associated with increased inflammation, including obesity and psychosocial stress – may represent one mechanism of altered basal ganglia function leading to symptoms of fatigue in patients with CFS. Given mechanistic data suggesting that altered dopamine availability may be a consequence of inflammatory effects on the brain, our findings suggest that a good avenue for future studies might be exploring whether drugs that increase dopamine availability in the brain would reduce symptom expression in some CFS patients who exhibit basal ganglia changes and/or increased inflammation.

There are several strengths and weaknesses of this study which should be considered in the interpretation of the data. First, the sample size of CFS subjects was relatively small. Although there were many more CFS subjects identified by our initial sampling strategy, we adhered to strict inclusion and exclusion criteria to limit the potential influence of factors such as psychotropic medications and psychiatric conditions like depression, both of which can have a profound effect on neuroimaging data. Moreover, we used validated assessment tools to quantify the degree of pathology in each of the domains relevant to the diagnosis of CFS. This attempt to provide a relatively homogenous sample of CFS patients also runs the risk of producing results that are not generalizable to the “typical” CFS patient. In addition, these data represent averaged results from each of the samples, and observation of the data indicates there was significant overlap between the groups. Thus, not all persons in our study exhibited reduced neural activation in the basal ganglia, although all CFS subjects exhibited significant fatigue. Therefore, although alterations in basal ganglia function may characterize a subgroup of CFS patients, such alterations do not typify all CFS subjects and do not account for all instances of fatigue. In this study non-fatigued controls were used as the comparison group as a starting place in investigating biologic associations with CFS. These non-fatigued controls exhibited some low level of minor medical problems including allergies, muscle aches and pains, gastrointestinal distress, high blood pressure and hypercholesterolemia for which they were taking medications, but not at rates that differed from our CFS sample. Nevertheless, in order to evaluate whether the findings are specific to CFS, additional studies comparing persons with CFS to persons with more significant illnesses or chronic fatigue would be useful to determine if our findings are typical of CFS patients, or patients with fatigue or illness in general. Finally, it should be noted that the CFS patients in this study were not drawn from a sample of subjects seeking care at an academic or private practice specialty clinic providing tertiary care. Rather the subjects studied herein were a community-based sample, and despite the rigorous inclusion and exclusion criteria, might more likely reflect findings in the average patient.

## Conclusions

CFS subjects free of psychotropic medications and without significant psychiatric disease were found to exhibit reduced activation of basal ganglia structures that correlated with symptoms of fatigue. The findings are consistent with diseases known to affect the basal ganglia and are consistent with basal ganglia changes seen after administration of a variety of stimuli that induce an inflammatory response.
